# Inhibition of autophagy potentiates the efficacy of Gli inhibitor GANT-61 in MYCN-amplified neuroblastoma cells

**DOI:** 10.1186/1471-2407-14-768

**Published:** 2014-10-17

**Authors:** Jing Wang, Song Gu, Jun Huang, Sheng Chen, Zhen Zhang, Min Xu

**Affiliations:** Department of Surgery, Shanghai Children’s Medical Center, Shanghai Jiaotong University School of Medicine, Shanghai, 200127 China; Hematology and Oncology Department, Shanghai Children’s Medical Center, Shanghai Jiaotong University School of Medicine, Shanghai, 200127 China; Institute for Pediatric Translational Medicine, Shanghai Children’s Medical Center, Shanghai Jiaotong University School of Medicine, Shanghai, 200127 China; Shanghai Pediatric Congenital Heart Disease Institute, Shanghai Children’s Medical Center, Shanghai Jiaotong University School of Medicine, Shanghai, 200127 China

**Keywords:** Neuroblastoma, GANT-61, MYCN amplification, Autophagy

## Abstract

**Background:**

Aberrant Hedgehog (Hh) signaling is often associated with neuroblastoma (NB), a childhood malignancy with varying clinical outcomes due to different molecular characteristics. Inhibition of Hh signaling with small molecule inhibitors, particularly with GANT-61, significantly suppresses NB growth. However, NB with MYCN amplification is less sensitive to GANT-61 than those without MYCN amplification.

**Methods:**

Autophagic process was examined in two MYCN amplified and two MYCN non-amplified NB cells treated with GANT-61. Subsequently, chemical and genetic approaches were applied with GANT-61 together to evaluate the role of autophagy in GANT-61 induced cell death.

**Results:**

Here we show that GANT-61 enhanced autophagy in MYCN amplified NB cells. Both an autophagic inhibitor 3-methyladenine (3-MA) and genetic disruption of ATG5 or ATG7 expression suppressed GANT-61 induced autophagy and significantly increased apoptotic cell death, whereas pre-treatment with an apoptotic inhibitor, Z-VAD-FMK, rescued GANT-61 induced cell death and had no effect on the autophagic process. In the other hand, GANT-61 barely induced autophagy in MYCN non-amplified NB cells, but overexpression of MYCN in MYCN non-amplified NB cells recapitulated GANT-61 induced autophagy seen in MYCN amplified NB cells, suggesting that the level of GANT-61 induced autophagy in NB cells is related to MYCN expression level in cells.

**Conclusion:**

Aberrant Hh signaling activation as an oncogenic driver in NB renders inhibition of Hh signaling an effective measure to suppress NB growth. However, our data suggest that enhanced autophagy concomitant with Hh signaling inhibition acts as a pro-survival factor to maintain cell viability, which reduces GANT-61 efficacy. Besides, MYCN amplification is likely involved in the induction of the pro-survival autophagy. Overall, simultaneous inhibition of both Hh signaling and autophagy could be a better way to treat MYCN amplified NB.

**Electronic supplementary material:**

The online version of this article (doi:10.1186/1471-2407-14-768) contains supplementary material, which is available to authorized users.

## Background

NB is one of the most common solid malignant tumors in children. It arises from neural crest element of the sympathetic nervous system and usually occurs in the adrenal medulla [1]. Nearly half neuroblastoma is classified as high-risk group due to aggressive tumor and poor prognosis. Many of them are associated with MYCN oncogene amplification [2]. Although children with high-risk tumor are aggressively treated with multi-modal therapy, this group still has poor survival rate (40%-50%) [3]. Therefore, it is critical to understand the underlying therapy-resistant mechanism of high-risk tumor.

The Hedgehog (Hh) signaling pathway plays a crucial role in the regulation of numerous embryonic development processes, including neural crest proliferation, differentiation and patterning [4]. Its signaling cascade is mainly comprised of Hh ligands [Sonic Hedgehog (Shh), Indian Hedgehog (Ihh) and Desert Hedgehog (Dhh)], receptors [patched (Ptch) and smoothened (Smo)], and downstream effectors (Gli1, 2 and 3) [5]. Aberrant activation of Hh signaling and ectopic expression of its components are observed in several malignancies such as medulloblastoma, basal cell carcinoma, rhabdomyosarcoma, etc [6, 7]. Inhibition of Hh signaling has been shown to be an effective way to block cancer cell proliferation and induce cell apoptosis [8, 9]. Two strategies have been used to block Hh signaling. One is to target the transmembrane receptor Smo with cyclopamine or SANT1 [10, 11], the other is to interfere with downstream effector Gli transcription factors with GANT-61 [12]. Given the position of Gli in Hh signaling cascade, GANT-61 has an advantage of functioning regardless of the mechanism of Hh signaling activation, which would be particularly useful for treating tumors with mutations constitutively activating Hh signaling upstream of Gli.

NB also has aberrant expression of Hh signaling components and is susceptible to Hh signaling inhibitor [13, 14]. Both cyclopamine and GANT-61 have been shown to suppress NB cell growth and induce apoptosis, with GANT-61 being more effective [15, 16]. However, similar to the reaction of NB cells to conventional chemotherapeutic drug, MYCN amplified NB cells are also more resistant to Hh signaling than non-MYCN amplified NB cells do.

Here we provide first evidence that GANT-61 can induce autophagy in MYCN amplified NB cell lines. Inhibition of autophagy increased cell death though apoptosis, suggesting a pro-survival role of autophagy in MYCN amplified NB cell lines. However, inhibition of apoptosis did not change the level of autophagy, although it rescued cell viability. It indicates that induction of autophagy may be an intrinsic property of GANT-61 for treating MYCN amplified NB cells. Furthermore, we found that the autophagic level in MYCN non-amplified NB cells was hardly affected by GANT-61, but MYCN overexpression in MYCN non-amplified NB cells could enhance GANT-61 induced autophagy, suggesting that MYCN amplification has a positive role in the induction of the pro-survival autophagy. Our data point out that a combination of GANT-61 and autophagy inhibitor may be a good approach to treat high-risk NB with MYCN amplification.

## Methods

### Chemicals

GANT-61, 3-Methyladenine (3-MA) and Z-VAD-FMK were purchased from Sigma Chemical Co (St. Louis, MO, USA). Bafilomycin A1 (BafA1) was purchased from Cayman Chemical (Ann Arbor, MI, USA).

### Cell culture

Four human neuroblastoma cell lines were used in our research. SK-N-BE(2) cells and SH-SY5Y cells were purchased from the Shanghai Institute of Cell Biology, Chinese Academy of Sciences (Shanghai, China). NBL-W-S and SK-N-AS cells were obtained from the State Key Laboratory of Hematology Oncology of the Ministry of Health, Shanghai Children’s Medical Center (Shanghai, China). SK-N-BE(2), SH-SY5Y and NBL-W-S cells were maintained in DMEM (Dulbecco’s Modified Eagle Medium) with high glucose (Life Technologies, Grand Island, NY, USA). SK-N-AS cells were cultured in DMEM with low glucose (Life Technologies, Grand Island, NY, USA). Both media were supplemented with 10% FBS (Life Technologies, Grand Island, NY, USA) and 1% penicillin/streptomycin (Sigma-Aldrich Co, St. Louis, MO). Cells were cultured as monolayer in a humidified atmosphere containing 5% CO_2_ at 37°C. When cells reached a confluence of 60-80%, media were switched to serum-free media for drug treatment.

### Fluorescence in situ hybridization

The hypotonic solution was preheated at 37°C for 30min. NB cells were precipitated, put into the pre-warmed hypotonic solution, concentrated, fixed and then dropped onto clean microscope slides. Slides were allowed to dry at 56°C for 1h or at room temperature overnight. Denaturation and hybridization were done with LSI MYCN Probe mixture (Vysis, Downers Grove, IL) as described by the manufacturer. DAPI II counterstain (Vector Laboratories, Burlingame, CA) was added to visualize nucleus and slides were incubated in the dark for 30 min at 4°C before imaging. Images were taken under an upright fluorescence microscope (OlympusIX51, Japan).

### Cell viability assay

Cell viability and cytotoxicity was assessed by MTT [3-(4,5-dimethylthiazol-2-yl) -2,5-diphenylterazo-lium] colorimetric assay (Sigma, Steinheim, Germany). Cells were seeded in 96-well plates at the density of 10,000 cells in 100 μL medium per well. MTT solution was added to the culture medium (final concentration 500 μg/ml) and incubated for 4h at 37°C in the dark. After incubation, the supernatant was aspirated and formazan crystals were dissolved in 100μL of DMSO at 37°C for 15 min with gentle agitation. The absorbance value at 570 nm was read using Glo Max-Multi Detection System (Promega, USA). Data was analyzed from three independent experiments and was normalized to the absorbance of wells containing media only (0%) and untreated cells (100%). IC50 values were calculated from Sigmoidal dose-response curves using Sigmaplot 10.0 software.

### Protein extraction and Western blot analysis

Cells were washed twice with cold PBS (phosphate buffer saline) and were harvested by trypsinization. For total protein isolation, cells were suspended in cell lysis buffer (Beyotime, China) and incubated on ice for 30 min. The suspension was collected after centrifugation at 15000 g for 15min at 4°C. Protein concentrations were measured using the BCA protein assay kit (Bio-Rad, Hercules, CA, USA) according to the manufacturer’s instruction. Fifty microgram of proteins were denatured by boiling at 96°C for 5 min in sample buffer (0.5M Tris-HCL, PH 6.8, 4%Sodium dodecyl sulfate, 20% glycerol, 0.1% bromphenol blue, 10% β-mercaptoethanol) in a ratio of 1:1. Equivalent amounts of protein were loaded and electrophoresed in 6-15% SDS (Sodium dodecyl sulfate)-PAGE gels. After electrophoresis, gels were transferred to nitrocellulose (NC) filter membranes or polyvinylidene fluoride (PVDF) membranes. Membranes were blocked with blocking buffer for 1h and then were incubated with various primary antibodies with 1:1000 dilution for 2 h. After washing with PBS, membranes were incubated with IRDye 800-conjugated goat anti-rabbit IgG secondary antibody (Rockland, USA) with 1:2000 dilution for half an hour at room temperature. Immunoreactive bands were visualized with a Licor Odyssey Infrared Imaging System (LI-COR Biosciences, USA). Primary antibodies against ATG5, ATG7, BECLIN-1, cleaved CASPASE3, AKT and phosphorylated AKT (ser473) and β-ACTIN were purchased from Cell Signaling Technology, Inc. (Danvers, MA, USA). LC3B were purchase from Novus Biologicals (Littleton, CO, USA) and BCL-2 was purchased from Santa Cruz Biotechnology (Santa Cruz, CA, USA). All the Western blot experiments were reproduced in three separate experiments

### Annexin V binding assay

The Annexin V binding assay was performed according to manufacturer’s instruction using Annexin V-FITC or Annexin V-PE detection kit I (BD Biosciences, San Diego, CA, USA). Cells were harvested by trypsinization and washed twice with cold PBS. Cell pellets were re-suspended with 100 μL binding buffer at a density of 1×10^5^ cells per ml and incubated with 5μL of FITC or PE-conjugated AnnexinV and 5 μL of propidium iodide (PI) or 7-AAD for 15 min at room temperature in the dark. 400 μl of 1X binding buffer was added to each sample tube, and immediately the samples were analyzed by BD FACSCanto II Flow Cytometer (BD Biosciences, USA).

### Acridine orange staining

Acridine orange (AO) (Sigma-Aldrich Co.) was used to evaluate and quantify the formation of acid vesicular organelles (AVOs) by fluorescence microscopy and flow cytometry. AO is an acidotropic fluorescent dye that stain DNA and cytoplasm bright green. When protonated in the presence of acid compartments, AO fluorescences bright red. Cells were treated with 200 nmol/l BafA1 for 30 min before adding AO to inhibit the acidification of autophagic vacuoles. After incubated with the BafA1, cells were treated with AO (1 μg/ml) in serum-free medium for 15 min at 37°C. AO was removed and fluorescent micrographs were obtained using an inverted fluorescence microscope (Leica DMI3000, Solms, Germany). Green (510-530 nm) and red (650 nm) fluorescent emission from 1×10^4^ cells illuminated with blue (488 nm) excitation light was measured with a BD FACSCanto II Flow Cytometer (BD Biosciences, USA).

### Monodansylcadaverine (MDC) incorporation assay

Autophagic vacuoles were also detected with MDC staining. Cells were incubated with MDC (50 μM) in PBS at 37°C for 10min. After incubation, cells were washed four times with cold PBS and fixed with 3.75% paraformaldehyde in PBS. Cells were immediately analyzed under an inverted florescence microscope (Leica DMI3000, Solms, Germany).

### Detection and quantification of endogenous LC3 vacuoles

Cells were fixed in 3.7% paraformaldehyde (PFA) in PBS for 10 min at room temperature. Coverslips were washed in PBS and blocked with 1% bovine serum albumin (BSA)/PBS for 1 h. Next, cells were incubated with anti-LC3 antibody (Diluion 1:400, Novus Biologicals, USA) in 1% BSA/PBS for 2 h at room temperature. After washing 3 times with PBS, slides were stained for 1h with goat anti rabbit IgG-H&L Cy3 antibody (Abcam, Hongkong). Samples were mounted with 80% glycerol and photographed under an upright florescence microscope (Leica DM6000, Germany). Image analysis was done with Image-Pro Plus (Media Cybernetics, USA). The average number of fluorescent puncta in untreated NB cells is used a basal value for AVOs in untreated conditions. Cells with a value 5 times higher than the basal value were scored as cells with active autophagic process. The percentages of positive cells were determined by counting a total of more than 50 cells in each sample and 3 samples were analyzed in each group.

### Formation and quantification of exogenous GFP-LC3 vacuoles

Cells were transfected with GFP-LC3 plasmid (Gift of Dr. Zhixue Liu, Institute for Nutritional Sciences, SIBS, China), using Lipofectamine LTX (Life Technologies, USA) according to manufacturer’s instruction. After 10uM GANT-61 treatment for 48h, cells were fixed with 4% PFA in PBS, analyzed and photographed under an upright florescence microscope (Leica DM6000, Germany). For quantification of autophagic cells, the proportion of cells with punctate green dots were determined from triplicates.

### ATG5 and ATG7 shRNA knockdown

Lentiviral ATG5 and ATG7 shRNA vectors were purchased from (Genechem, China). Lentiviral particles were produced by calcium phosphate transfection of lentiviral shRNA vector, envelope vector PMD2.G and packaging vectors Δ8.74 into 293FT cells (the molar ratio of plasmids is 2:3:3). Supernatant with viral particle was collected 48 h after transfection. Viral transduction was facilitated with 8μg/ml polybrene. shRNA sequences listed below: ATG5: 5′-TTCATGGAATTGAGCCAAT-3′; ATG7: 5′-TTTGGGATTTGACACATTT-3′; scramble control: 5′- TTCTCCGAACGTGTCACGT-3′.

### MYCN overexpression

MYCN expression vector (pCMV6-XL4-MYCN) was purchased from (OriGene Technologies, Inc, USA). Plasmid DNA was prepared from Endofree Plasmid Kit (Tiangen, China) and transfected into NB cells through electroporation (Nepa Gene, Japan). Cells were trypsinized and resuspended in Opti-MEM (Life Technologies, USA). 10 μg DNA was mixed with 1×10^6^ cells in a volume of 100μl cells and placed into an electroporation cuvette. Place the cuvette with cell-DNA mixture in the electroporation holder. Set the electroporator at 150 V and pulse cells twice with a pulse width of 5ms and an interval of 50ms. After electroporation, cells were quickly transferred into a pre-warmed culture plate containing media for the following experiments.

### Statistical analyses

Results were presented as means ± standard deviation (SD). The significance of difference between groups was determined by Student’s *t*-test.

## Results

### GANT-61 decreases cell viability and induce apoptosis in MYCN amplified NB cells

**Figure 1:**
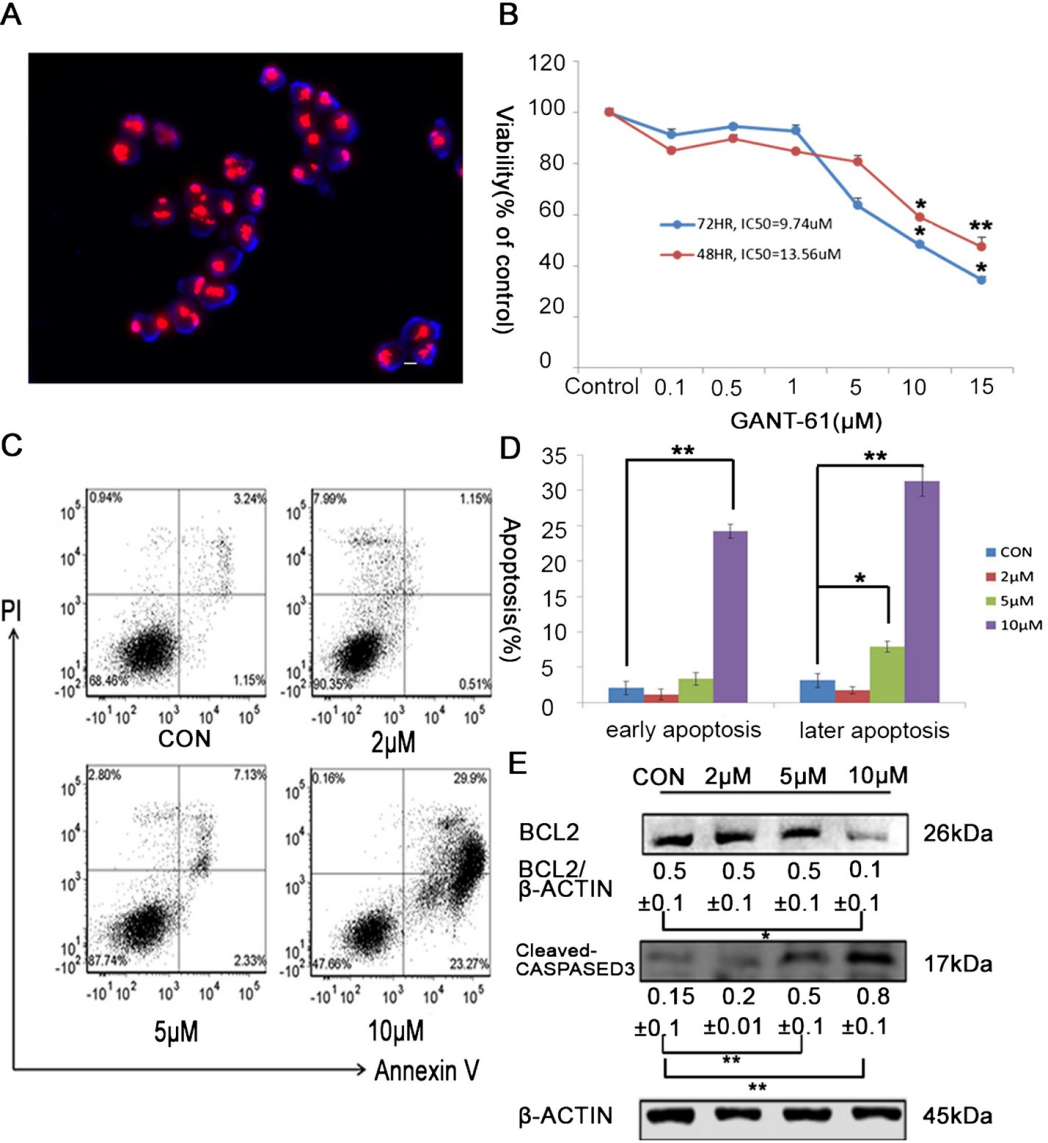
**GANT-61 induces cell cytotoxicity and apoptosis in NB cells. (A)** FISH analysis of MYCN amplification in NBL-W-S cells. Scale bar, 10μm. **(B)** NBL-W-S cells were treated with GANT-61 at various concentration (0.1-15μM) for indicated times and the cytotoxicity was measured by MTT assay. The percentage of viable cells was calculated as a ratio of treated to control cells. Data is expressed as the mean ± SD of three independent experiments. *P < 0.05, **P < 0.01. **(C)** Representative flow cytometry analysis of apoptosis after Annexin V and PI-double staining. NBL-W-S cells were treated with the indicated concentration of GANT-61 for 48h. **(D)** Histogram of flow cytometry analyses from 3 independent experiments. *P < 0.05, **P < 0.01. **(E)** Western blot analysis was carried out to detect the expression of apoptosis-related proteins. NBL-W-S cells were treated with indicated concentrations of GANT-61 for 48h. The BCL2/β-ACTIN and Cleaved-CASPASE3/β-ACTIN ratios were determined by densitometry (mean ± SD), *P < 0.05, **P < 0.01. The expression of anti-β-ACTIN is used as a loading control. CON, control.

GANT-61, an inhibitor of Hh signaling effector GLI protein, was reported to have a cytotoxic effect on NB cell lines, such as SK-N-AS, SH-SY5Y, SK-N-DZ etc., and GANT-61 efficacy is reversely correlated with MYCN expression [16]. To examine whether the reduced GANT-61 sensitivity is a general phenomenon for MYCN amplified NB cells. We tested the effect of GANT-61 on two other NB cell lines, NBL-W-S and SK-N-BE(2), which have multiple-copy MYCN amplification (Figure 1A and Additional file 1: Figure S1A). Using MTT assay, we examined the viability of NB cells under various concentrations of GANT-61 (0.1-15 μM) at 48 h and 72 h after drug treatment. The low concentrations of GANT-61 did not substantially affect cell viability, while the higher concentrations of GANT-61 significantly reduced the number of NB cells. SK-N-BE(2) cells had fewer viable cells at high concentration of GANT-61, with 11.52% live cells at 72 h under 15 uM GANT-61, whereas NBL-W-S cells had 34.43% live cells under the same condition. The IC_50_ values of GANT-61 for NBL-W-S cells at 48 h and 72 h after treatment are 13.56 μM and 9.74 μM, respectively (Figure 1B). Similar values were also achieved for SK-N-BE(2) cells. The IC_50_ values of GANT-61 at 48 h and 72 h after treatment are 10.9 μM and 7.96 μM, respectively (Additional file 1: Figure S1B). Both IC_50_ values at 72 h are similar to those reported for other MYCN amplified NB cells [16], indicating that it is quite a general rule for GANT-61 to have a lower cytotoxic effect on MYCN amplified NB cells. Since 48 h treatment of GANT-61 already had significant effect on both cell lines, we used 48 h treatment of 10uM GANT-61 for all the following experiments, if not indicated otherwise.

Previous studies have shown that cytotoxicity of GANT-61 is mediated though apoptosis. Therefore, we used Annexin V-FITC/PI double staining to evaluate apoptotic status of NB cells after GANT-61 treatment. Indeed, the number of apoptotic cells increased with the concentration of GANT-61 in both NB cell lines (Figure 1C and Additional file 1: Figure S1C). The sum of early apoptotic cells (PI-/Annexin-FITC+) and late apoptotic/necrotic cells went up to around 50% in both cell lines (Figure 1C-1D and Additional file 1: Figure S1C-1D). Interestingly, we noticed a small population of necrotic cells existed even in untreated NB cells. However, the percentage of this population in the whole population didn’t change too much with the increased GANT-61 concentration.

To examine the biochemical changes in the GANT-61 induced apoptosis, we measured the expression of critical regulators of apoptotic process by Western blot. GANT-61 significantly decreased the expression of apoptotic regulator BCL2 and increased the level of active CASPASE3 in both NB cell lines (Figure 1E and Additional file 1: Figure S1E), suggesting the intrinsic apoptotic pathway plays a major role in GANT-61 induced apoptosis.

### GANT-61 induces autophagy in NB cells

**Figure 2:**
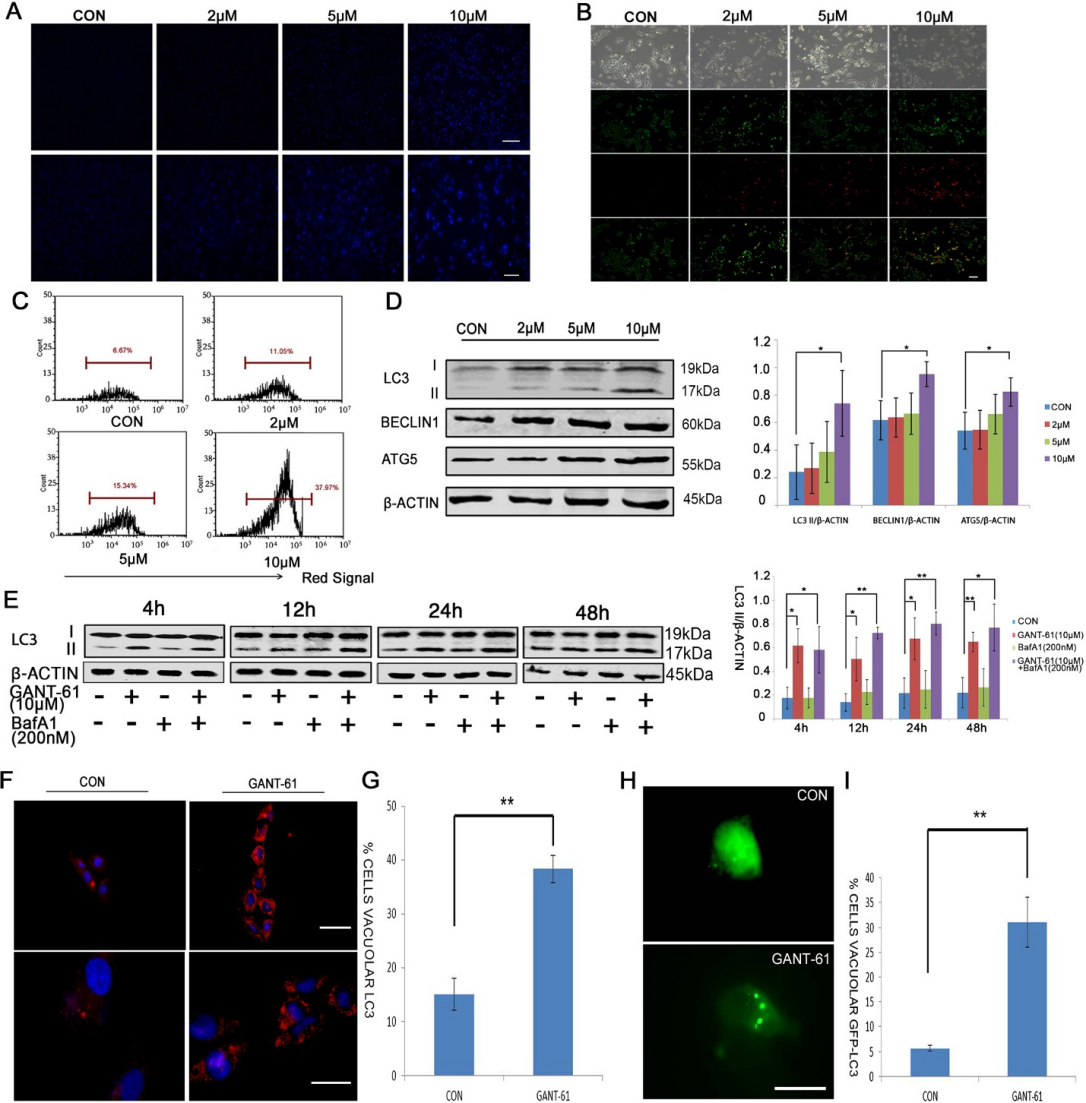
**GANT-61 induces autophagy in NB cells. (A)** MDC staining revealed that autophagy was activated in NBL-W-S cells after 48h treatment with GANT-61. Scale bars, top: 100μm, bottom: 50μm. **(B)** Fluorescence microscopy of AO stained NBL-W-S cells treated with the indicated drug for 48h. Top row, phase contrast images; Second row, images captured through green fluorescence filter; Third row, images captured through red fluorescence filter; Bottom row, merged images. Scale bars, 100μm. **(C)** Flow cytometry analysis of AO stained cells in panel B. **(D)** The expression of autophagic proteins in NBL-W-S cells treated with various concentrations of GANT-61 for 48h. The densitometry ratios of LC3 II/β-ACTIN**,** ATG5/β-ACTIN and BECLIN1**/**β-ACTIN were plotted as histogram (mean ± SD), *P < 0.05. **(E)** Effect of lysosomal inhibitor BafA1 on autophagic flux induced by GANT-61. LC3 immunoblotting was performed to evaluate LC3 conversion. NBL-W-S cells were first treated with 200nM BafA1 for 30 min and then treated with 10μM GANT-61 for 4h, 12h, 24h or 48h. The LC3 II/β-ACTIN ratio at different time points was plotted as histogram (mean ± SD), *P < 0.05, **P < 0.01. **(F)** Immunofluorescence with LC3 antibody on NBL-W-S cells after 48h GANT-61 treatment. Scale bars, top: 500μm, bottom: 20μm. CON, control. **(G)** Quantification of cells with a number of LC3 puncta five times higher than basal level in panel F, **P < 0.01. **(H)** NBL-W-S cells transfected with GFP-LC3 plasmids were treated with GANT-61 for 48h. A puncta pattern of GFP-LC3 was formed after drug treatment. Scale bar,20μm. **(I)** Quantification of cells with GFP-LC3 puncta in panel H, **P < 0.01. Equal loading and transfer were verified by re-probing membranes with anti-β-ACTIN antibody in Western blot analysis.

Numerous studies have shown that autophagy has a dual role in tumorigenesis, either functioning as a tumor suppressor though removing aberrant proteins and organelles, or as a mechanism maintaining cell survival under stress [17–19]. To understand whether autophagy has a role in GANT-61 induced cell death, we examined the formation of autophagic vacuoles with MDC staining. MDC is an autofluorescent compound that preferentially accumulates in mature autophagic vacuoles, such as autophagolysosomes. As shown in Figure 2A and Additional file 2: Figure S2A, we noticed a gradual increase in the number and fluorescent intensity of MDC-labeled vesicles with an increasing GANT-61 concentration in both NB cell lines. It suggests an induction of autophagic vacuoles formation by GANT-61.

We further validated this finding with other assays. AO is widely used for detection of AVOs, an indicator of autophagy. In acid compartments, such as lysosomes and autolysosomes, the fluorescence of AO switches from green to red color. Consistent with MDC staining result, we observed an increasing red fluorescence with the increasing GANT-61 concentration, while control cells primarily exhibited green fluorescence with minimal red fluorescence (Figure 2B and Additional file 2: Figure S2B). Using flow cytometry to do quantitative analysis, we observed five- and two-fold increase of red fluorescent signal in NBL-W-S and SK-N-BE(2) cells, respectively (Figure 2C and Additional file 2: Figure S2C).

A symbolic marker of autophagosome formation is the conversion of a soluble form of LC3-I to autophagic vesicle-associated form LC3-II. We did note a significantly increased LC3-II level after GANT61 treatment (Figure 2D and Additional file 2: Figure S2D), suggesting an enhanced autophagic induction with the increasing GANT-61 concentration. Consistently, the level of BECLIN1, a critical component for autophagosome initiation, significantly increased with the increasing GANT-61concentration (Figure 2D and Additional file 2: Figure S2D). The level of another autophagosomal component ATG5 also significantly increased in GANT-6-treated NBL-W-S cells (Figure 2D), though no significant increase was observed in GANT-6-treated SK-N-BE(2) cells (Additional file 2: Figure S2D).

To determine whether GANT-61 induces autophagic production or disrupt autophagosomal consumption, we applied a lysosomal proton pump inhibitor Bafilomycin A1 (BafA1) to block the autophagosome-lysosome fusion step. Cells were collected for anti-LC3 immunoblotting at 4 h, 12 h, 24 h and 48 h after GANT-61 treatment. From 12h on, cells treated with both GANT-61 and BafA1 started to show a higher level of LC3-II than cells treated with GANT-61 only (Figure 2E and Additional file 2: Figure S2E), indicating that GANT-61 stimulates autophagosomal production rather than autophagosomal sysnthesis.

Autophagosomal formation was also visually examined by immunofluorescence in both cell lines. GANT-61 significantly increased endogenous LC3 puncta in both MYCN amplified cell lines (Figure 2F-2G and Additional file 2: Figure S2F-S2G). In addition, We transfected both NBL-W-S and SK-N-BE(2) cells with GFP-LC3 plasmid to monitor autophagic induction. After GANT-61 treatment, we observed a distribution pattern change from a homogenous distribution into punctuate dots in the cytoplasm of a significant amount of cells (Figure 2H-2I and Additional file 2: Figure S2H-S2I), indicating an increased autophagosomal formation in these cells.

Overall, our fluorescent microscope and biochemical data indicate that GANT-61 can induce autophagic induction in MYCN amplified NB cells.

### Induced autophagy after GANT-61 treatment is pro-survival

**Figure 3:**
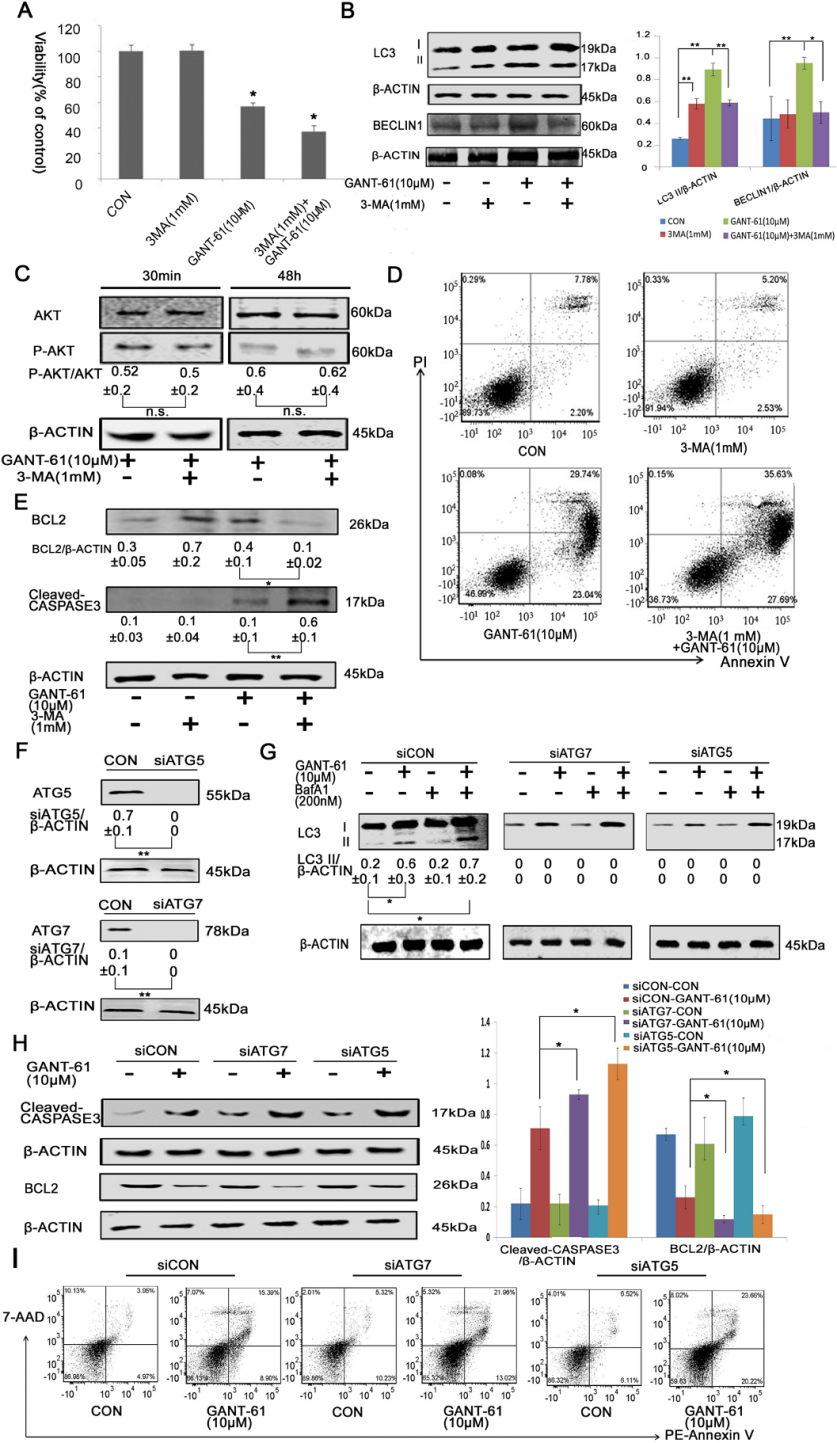
**Effects of autophagic inhibition on GANT-61 treated NB cells. (A)** 3-MA pre-treatment enhanced GANT-61 toxicity on NBL-W-S cells. Cell viability was measured by MTT assay. **(B)** The effect of 3-MA on the expression of autophagic proteins in NBL-W-S cells. Western blot analysis was performed with anti-LC3, anti-BECLIN-1 and anti-ATG5 antibodies. The densitometry ratios of LC3 II/β-ACTIN, BECLIN1/β-ACTIN and ATG5**/**β-ACTIN were plotted as histogram. **(C)** 3-MA pre-treatment did not affect the level of AKT phosphorylation in NBL-W-S cells after GANT-61 treatment. Values of P-AKT/AKT ratio were listed under p-AKT blots. **(D)** 3-MA increased cell apoptosis in GANT-61 treated NBL-W-S cells. Apoptotic cells were analyzed using Annexin V/PI double staining. **(E)** The effect of 3-MA on apoptotic protein expression. Western blot analysis was performed with anti-BCL-2 and anti-cleaved CASPASE3 antibodies. The BCL2/β-ACTIN and Cleaved-CASPASE3/β-ACTIN ratios were listed under blots. **(F)** Knockdown of essential autophagic component ATG5 or ATG7 by shRNA in NBL-W-S cells was verified by Western blot analysis with anti-ATG5 or anti-ATG7 antibodies. The ATG-5/β-ACTIN and ATG-7/β-ACTIN ratios were listed under blots. **(G)** Knockdown of ATG5 or ATG7 completely abolished GANT-61 induced LC3 conversion, even in the presence of lysosomal inhibitor BafA1. Knockdown cells were first treated with 200nM BafA1 for 30 min and then treated with 10μM GANT-61 for 12h. The LC3 II/β-ACTIN ratio was listed under blots. **(H)** A higher level of cleaved CASPASE3 and a lower level of BCL2 were detected in ATG5 or ATG7 knockdown NBL-W-S cells. The BCL2/β-ACTIN and cleaved-CASPASE3/β-ACTIN ratios were plotted as histogram. **(I)** Representative flow cytometry analysis of apoptosis in GANT-61 treated cells. siCON: scramble shRNA knockdown control, siATG5: ATG5 shRNA knockdown, siATG7: ATG7 shRNA knockdown cells. CON, control. Data are expressed as the mean ± SD, *P < 0.05, **P < 0.01, n.s., no statistical significance.

Having known autophagosome formation was enhanced after GANT-61 treatment, we used autophagy inhibitor 3-MA to understand the role of autophagy in GANT-61 induced cell death. Incubation with 1mM 3-MA for 6h, a treatment commonly used to inhibit autophagy in cells, didn’t affect the viability of NB cell lines (Figure 3A and Additional file 3: Figure S3A). However, it significantly caused more cell death in GANT-61 treated cells (Figure 3A and Additional file 3: Figure S3A), suggesting a protective role of autophagy in GANT-61 induced cell death. As expected, the inhibition of GANT-61 induced autophagy by 3-MA pre-treatment was evidentiated by the decreased levels of autophagosome markers LC3-II and BECLIN 1 (Figure 3B and Additional file 3: Figure S3B), possibly through a feedback elicited from autophagic inhibition.

Since 3-MA is shown to have non-specific effects other than autophagy inhibition in different cellular environments, such as inhibition of class I PI3K that could lead to suppression of AKT phosphorylation [20], the increased cell death caused by 3-MA pre-treatment might be mediated through inhibition of AKT pro-survival pathway instead of autophagy inhibition. To exclude this possibility, we examined AKT phosphorylation at 30 min and 48 h after GANT-61 treatment. In our experimental condition, 3-MA pre-treatment didn’t change the level of AKT phosphorylation in GANT-61 treated cells at both time points (Figure 3C and Additional file 3: Figure S3C). It suggests autophagic induction is likely the major mechanism that MYCN amplified NB cells use to resist the cytotoxicity of GANT-61.

To check whether the additional cell death was caused by apoptosis, Annexin V-FITC/PI staining was applied to quantitate apoptotic cells. Indeed, the combination of 3-MA and GANT-61 increased the apoptotic cells from 52.78% and 54.11% up to 63.32% and 61.47% in NBL-W-S and SK-N-BE(2) cell lines, respectively (Figure 3D and Additional file 3: Figure S3D). Our data also showed a significantly decreased level of BCL2 and an increased level of cleaved CASPASE3 in 3-MA pre-treated NB cells lines (Figure 3E and Additional file 3: Figure S3E), which again suggested that an increased cell death induced by GANT-61 in 3-MA pre-treated cells was caused by enhanced apoptosis.

More specifically, we used a genetic approach to disrupt the autophagic production. As shown in Figure 3F and Additional file 3: Figure S3F, ATG5 or ATG7 shRNA specifically knocked down ATG5 or ATG7 expression, respectively. Knockdown of essential autophagic components ATG5 or ATG7 completely abolished GANT-61 induced LC3 conversion (Figure 3G and Additional file 3: Figure S3G), indicating an autophagic inhibition. Similar to the effect of 3-MA pre-treatment, ATG5 knockdown decreased the level of BCL2 and increased the level of cleaved CASPASE3 in GANT-61 treated cells (Figure 3H and Additional file 3: Figure S3H), suggesting an enhanced cell apoptosis after autophagic inhibition. ATG7 knockdown in NBL-W-S cells also showed a significant alteration in response to GANT-61 treatment (Figure 3H), albeit a non-significant change was observed in ATG7 knockdown SK-N-BE(2) cells (Additional file 3: Figure S3H). Next, we used PE-Annexin V/7-AAD double staining to quantitate apoptotic cell death in GANT-61 treated shRNA knockdown cells. Indeed, GANT-61 caused more apoptotic cell death in both ATG5 and ATG7 knockdown cells than those in scramble shRNA knockdown cells (Figure 3I and Additional file 3: Figure S3I). These date further confirmed that autophagy is a protective mechanism preventing MYCN amplified NB cells from apoptotic cell death under the stress of GANT-61.

**Figure 4:**
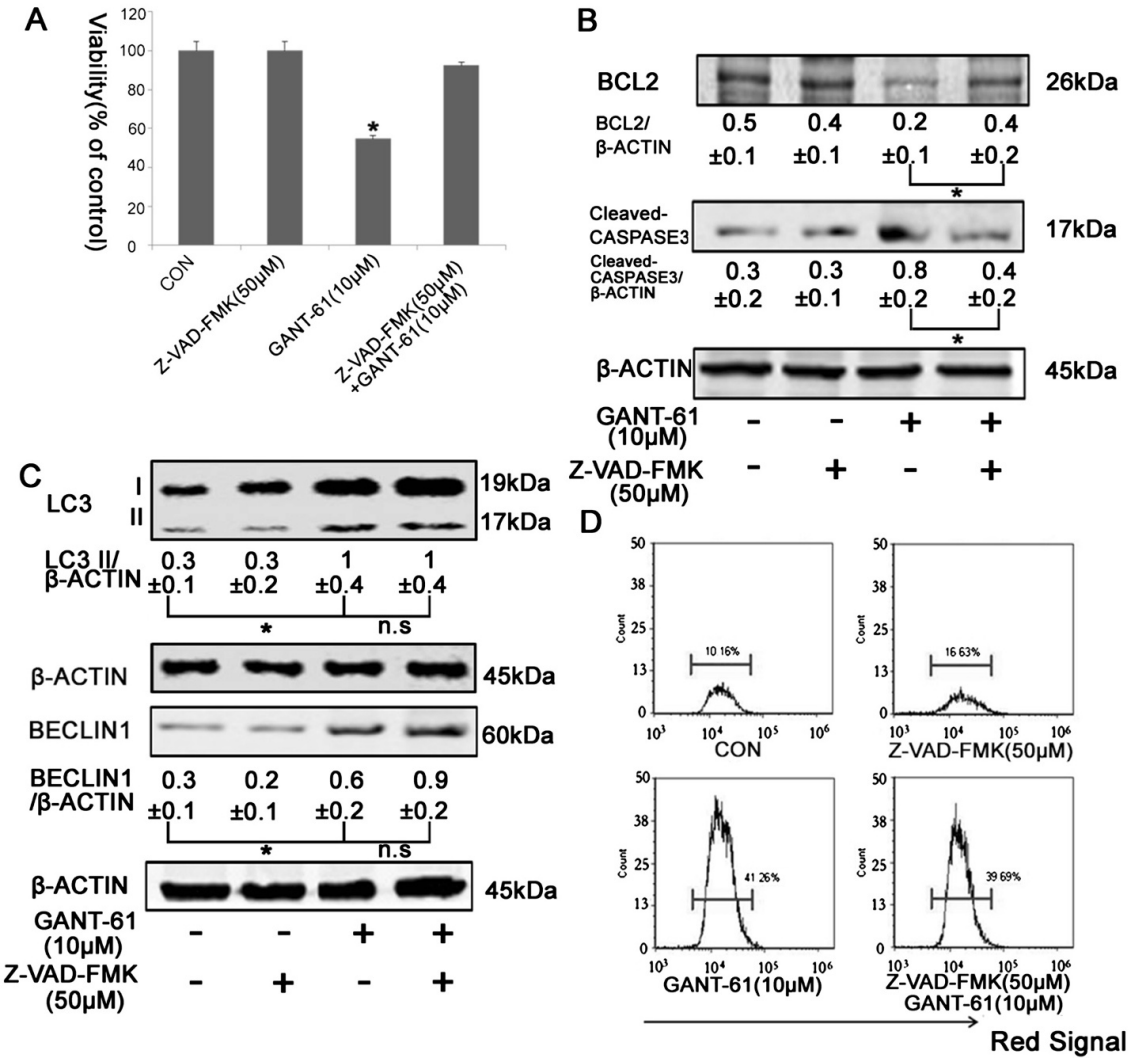
**Effects of an apoptotic inhibitor on GANT-61 treated NB cells. (A)** NBL-W-S cells were pre-treated with Z-VAD-FMK (50 μM) for 1h and then with 10uM GANT-61 for 48 h. Cell viability was measured by MTT assay. Data are expressed as the mean ± SD of three independent experiments. *P < 0.05. **(B)** The inhibitory effects of Z-VAD-FMK on apoptosis were detected by Western blot analysis with anti-BCL-2 and anti-cleaved CASPASE3 antibodies. The BCL2/β-ACTIN and cleaved-CASPASE3/β-ACTIN ratios were listed under blots (mean ± SD), *P < 0.05. **(C)** The expression of autophagic proteins was analyzed by Western blot with anti-LC3 and anti-BECLIN-1 antibodies. The densitometry ratios of LC3 II/β-ACTIN and BECLIN1**/**β-ACTIN were listed under blots, (mean ± SD), *P < 0.05, n.s., no statistical significance. **(D)** Flow cytometry histogram of AO stained cells treated with the indicated drug. CON, control. Equal loading and transfer were verified by re-probing membranes with anti-β-ACTIN antibody in Western blot analysis.

Since autophagy could also be pro-death, we asked whether inhibition of apoptosis in GANT-61 treated cells could affect cell viability or not. We used Z-VAD-FMK, an inhibitor of pan caspases, to suppress apoptotic pathway. First, we tested the cytotoxicity of Z-VAD-FMK in both NB cell lines and found no toxic effect in NBL-W-S and SK-N-BE(2) at the concentration of 50μM (Figure 4A and Additional file 4: Figure S4A). Therefore, we chose 50uM Z-VAD-FMK for apoptotic inhibition experiment. We observed that 50uM Z-VAD-FMK fully rescued cell viability after GANT-61 treatment (Figure 4A and Additional file 4: Figure S4A). A significantly increased level of BCL2 and a significantly decreased level of cleaved CASPASE 3 were also seen in Z-VAD-FMK treated cells (Figure 4B and Additional file 4: Figure S4B), verifying the occurrence of apoptotic inhibition. Interestingly, we noted that there was no change in the level of LC3-II and BECLIN 1 after addition of Z-VAD-FMK (Figure 4C and Additional file 4: Figure S4C). AO staining also revealed similar amounts of cells emitting red fluorescence between GANT-61 and GANT-61 plus Z-VAD-FMK groups (Figure 4D and Additional file 4: Figure S4D). Both indicate that apoptotic inhibition doesn’t affect GANT-61 induced autophagic process. These data again suggest that autophagy doesn’t function as an alternative cell death pathway in GANT-61 treated cells. Instead, it is a pro-survival factor to prevent cell death though apoptotic pathway triggered by GANT-61.

### The level of GANT-61 induced autophagy is lower in MYCN non-amplified NB cells

**Figure 5:**
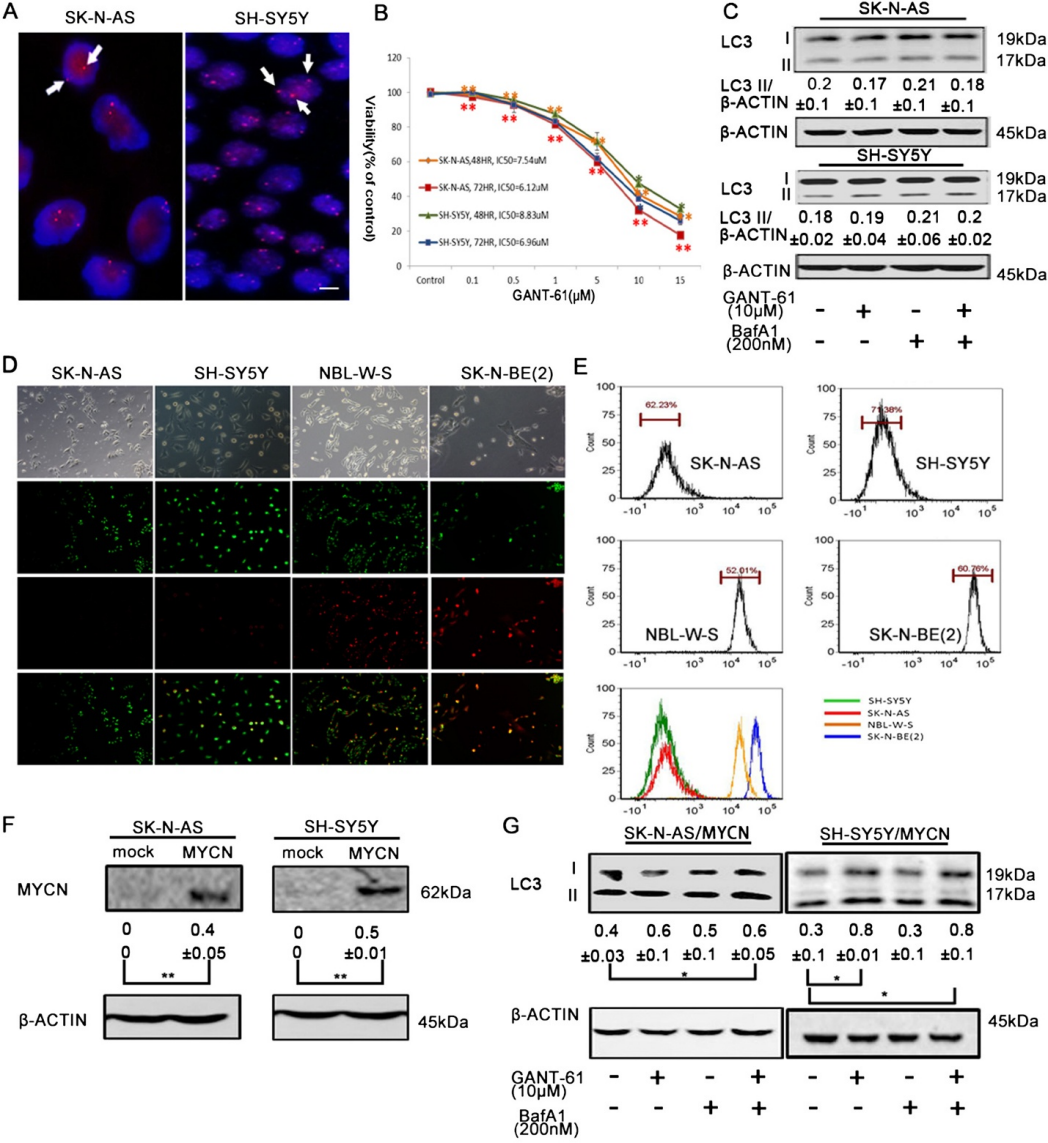
**GANT-61 induces a lower level of autophagy in MYCN non-amplified NB cells. (A)** FISH analysis of MYCN copy number in SK-N-AS and SH-SY5Y cells. White arrows indicate positive signals. Scale bars, 10μm. **(B)** SK-N-AS and SH-SY5Y cells were treated with GANT-61 at various concentration (0.1-15μM) for indicated times and the cytotoxicity was measured using a MTT assay. The percentage of viable cells was calculated as a ratio of treated to control cells. **(C)** The effect of lysosomal inhibitor BafA1 on LC3 conversion in SK-N-AS and SH-SY5Y cells treated with GANT-61. Cells were first treated with 200nM BafA1 for 30 min and then treated with 10μM GANT-61 for 12h. The LC3 II/β-ACTIN ratio was listed under blots. **(D)** Fluorescence microscopy of AO stained SK-N-AS, SH-SY5Y, NBL-W-S and NBL-W-S cells treated with 10μM GANT61 for 48h. Top row, phase contrast; Second row, green fluorescence; Third row, red fluorescence; Bottom row, merged images. Scale bars, 100μm. **(E)** Flow cytometry histograms of AO stained NB cells. The last plot summarizes four histogram profiles to visualize the difference of fluorescence intensities in MYCN non-amplified and amplified NB cells. **(F)** MYCN overexpression was verified by Western blot analysis with SK-N-AS and SH-SY5Y cells transfected with MYCN plasmid. The MYCN/β-ACTIN ratio was listed under blots. **(G)** The effect of lysosomal inhibitor BafA1 on LC3 conversion in SK-N-AS/MYCN and SH-SY5Y/MYCN cells treated with GANT-61. Cells were first treated with 200nM BafA1 for 30 min and then treated with 10μM GANT-61 for 12h. SK-N-AS/MYCN: SK-N-AS cells overexpressing MYCN. SH-SY5Y/MYCN: SH-SY5Y cells overexpressing MYCN. Equal loading and transfer were verified by re-probing membranes with anti-β-actin antibody. The LC3 II/β-ACTIN ratio was listed under blots. Data is expressed as the mean ± SD. *P < 0.05, **P < 0.01.

As MYCN non-amplified NB cells are more sensitive to GANT61 [16], we hypothesized that it is due to a lower level of the pro-survival autophagy induced in MYCN non-amplified NB cells. First, we tested the efficacy of GANT61 on SK-N-AS and SH-SY5Y cells, two known MYCN non-amplified NB cell lines (Figure 5A). Using MTT assay, we determined that the IC_50_ values of GANT-61 on SK-N-AS cells at 48 h and 72 h after drug treatment are 7.54 μM and 6.12 μM, respectively, and those on SH-SY5Y cells are 8.83 μM and 6.96 μM, respectively (Figure 5B). They are lower than the IC_50_ values of GANT-61 on NBL-W-S and SK-N-BE(2) (Figure 1A and Additional file 1: Figure S1A). It confirmed that MYCN non-amplified NB cells are more sensitive to GANT-61. Next, we examined the autophagic flux on these two MYCN non-amplified NB cell lines. Indeed, GANT-61 didn’t induce as much LC3 conversion in these two cell lines as it did in two MYCN amplified NB cell lines, even with the presence of lysosomal inhibitor BafA (Figure 5C, Figure 2E and Additional file 2: Figure S2E). It indicates that GANT-61 can’t enhance autophagic induction in GANT-61 treated MYCN non-amplified cells. Furthermore, we used AO for AVOs staining to evaluate the level of autophagy. Consistent with Western blotting results, we observed a weaker red fluorescence in both MYCN non-amplified cells (Figure 5D). Flow cytometry analysis quantitatively showed that the intensity of red fluorescence is much lower in both MYCN non-amplified cells than those in MYCN amplified cells (Figure 5E), suggesting a low level of autophagy in MYCN non-amplified cells. To recapitulate MYCN amplification in these MYCN non-amplified cells, we overexpressed MYCN in SK-N-AS and SH-SY5Y cells through electroporation (Figure 5F). It significantly enhanced autophagic production induced by GANT-61 (Figure 5G), demonstrating that MYCN amplification is a positive factor in GANT-61 induced protective autophagy.

## Discussion

NB is a common extra cranial tumor in childhood. Aberrant Hh signaling has recently been found associated with NB. Hh signaling inhibitors could suppress tumor growth through apoptotic induction [14, 16, 21]. Among them, GANT-61, a small molecular inhibitor of Hh signaling effector Gli, induces considerably greater cytotoxicity in NB cell lines than inhibitors of Hh signaling receptor Smo do. However, MYCN amplified NB cells are less susceptible to GANT-61 than MYCN non-amplified NB cells are [16]. We confirmed this observation with two additional MYCN amplified NB cell lines. MYCN amplification is so far the most reliable prognostic factor for high-risk NB [22, 23]. Our new findings reiterate clinical outcomes of high-risk NB with MYCN amplification.

MYCN is a transcription factor belonging to the MYC family of proto-oncogene, which regulates critical cellular processes including proliferation, cell growth and differentiation [24]. MYCN amplification is considered as an oncogenic driver of aggressive NB and MYCN targeted therapies have shown promises for treating this subset NB [25, 26]. Although numerous studies have focused on dissecting the molecular mechanism underlying MYCN-mediated resistance, a lot still remain to be discovered.

To understand the possible mechanism of GANT-61 resistance in MYCN amplified NB cell lines, we examined autophagic process in GANT-61 treated NB cells due to a role of MYC proteins in autophagic induction to promote cell survival [27]. Autophagy is a lysosome-dependent degradative pathway that is often induced under cell stress conditions to catabolize cytoplasmic contents [28–30]. It remains disputable whether therapy-induced autophagy in cancer cells is a self-defense mechanism to maintain cell survival or a non-apoptotic form of programmed cell death. Our data showed that GANT-61 could enhance autophagosomal formation in NB cells (Figure 2E and Additional file 2: Figure S2E), presenting an increasing number of AVOs stained by AO or MDC with an increasing drug concentration (Figure 2A-2C and Additional file 2: Figure S2A-S2C). Likewise, it was further evidentiated by increases in LC3-II levels and other autophagy-related molecules in GANT-61 treated NB cells (Figure 2D and additinoal Figure 2D). Consistently, activation of Shh signaling was shown to have a conserved role in suppressing autophagosomal synthesis from *drosophila* up to human, and both in normal and malignant cells, whereas inhibition of Shh signaling could increase autophagy [31, 32].

To understand the role of autophagy in GANT-61 induced cell death, autophagic formation was inhibited by 3-MA. We observed a significant increase of apoptotic cells after GANT-61 treatment (Figure 3D-3E and Additional file 3: Figure S3D-S3E). Using genetic approach, we confirmed this finding through knockdown of essential autophagic components ATG5 and ATG7 (Figure 3G-3I and Additional file 3: Figure S3G-S3I). These data demonstrate that autophagy induced by GANT-61 is indeed a protective factor in NB cells. Both GANT-61 and cyclopamine are small molecules inhibiting Hh signaling. But NB cells are more ressistant to cyclopamine [16]. Given the protective role of drug-induced autophage we showed here, cyclopamine resistance might be explained by a stronger autophagic induction, because it is shown that cyclopamine could not only increase autophagosomal synthesis through Smo acitivity inhibition, but also impair autophagosomal degradation through an unknow mechanism [31].

From the opposite end to approach this question, we inhibited apoptosis with Z-VAD-FMK. Inhibition of apoptosis rescued GANT-61 induced cell death, while no enhancement of autophagosomal formation was observed (Figure 4C and Additional file 4: Figure S4C). It indicates that GANT-61 induced autophagy is independent of cellular apoptotic process and is not a programmed cell death alternative to apoptosis.

It has been shown that both C-Myc and N-Myc overexpression are capable of inducing cytoprotective autophagy [27]. Several studies have also shown that tumors with high MYC expression activate autophagy to promote cell survival under drug treatment [32, 33]. Consistently, our results showed that GANT-61 treatment hardly induced LC3 conversion in MYCN non-amplified cells (Figure 5C). Ao staining showed much lower levels of red fluorescent autophagosome in GANT-61 treated MYCN non-amplified NB cells than those in GANT-61 treated MYCN amplified NB cells (Figure 5D), indicating that MYCN amplification is likely to have a role in GANT-61 induced autophagy in NB cells. Overexpression of MYCN in MYCN non-amplified NB cells indeed reiterate GANT-61 induced autophagy seen in MYCN amplified NB cells, which suggests that MYCN amplification could render NB cells the capability to resist GANT-61 toxicity through induction of pro-survival autophagy.

## Conclusions

Overall, our data revealed the existence of a protective autophagy in GANT-61 treated NB cells. The level of the pro-survival autophagy is related to MYCN expression level. Inhibition of autophagy in MYCN amplified NB cells could augment the efficacy of GANT-61 on MYCN amplified NB cells. It partially explains the GANT-61 resistance in MYCN amplified NB cells and suggests that a combination of GANT-61 and autophagic inhibitor could be a good approach to treat MYCN amplified NB. However, the molecular mechanism how MYCN amplification is involved in drug-induced autophagy remains to be answered.

## Electronic supplementary material

Additional file 1: Figure S1: GANT-61 induces cell cytotoxicity and apoptosis in NB cells**. (A)** MYCN amplification was evaluated by FISH analysis in SK-N-BE(2) cells. Scale bars, 10μm. **(B)** GANT-61 dosage response curves of SK-N-BE(2) cells were determined using a MTT assay. The percentage of viable cells was calculated as a ratio of treated to control cells. Data is expressed as the mean ± SD of three independent experiments. *P < 0.05, **P < 0.01. **(C)** Flow cytometry analysis of apoptosis after AnnexinV and PI-double staining SK-N-BE(2) cells were treated with indicated concentration of GANT-61 for 48h. **(D)** Histogram of flow cytometry analyses from 3 independent experiments. *P < 0.05, **P < 0.01, CON, control. **(E)** Western blot analysis was performed to detect the expression of apoptosis-related proteins. SK-N-BE(2) cells were treated with indicated concentration of GANT-61 for 48h. The BCL2/β-ACTIN and Cleaved-CASPASE3/β-ACTIN ratios were determined by densitometry (mean ± SD), *P < 0.05, **P < 0.01. Equal loading and transfer were verified by re-probing membranes with anti-β-ACTIN antibody. CON, control. (JPEG 520 KB)

Additional file 2: Figure S2: GANT-61 induces autophagy in NB cells. **(A)** MDC staining showed that the autophagy was activated in SK-N-BE(2) cells after GANT-61 treatment for 48h. Scale bars, top: 100μm, bottom: 50 μm. **(B)** Fluorescence microscopy of AO stained SK-N-BE(2) cells treated with the indicated concentration of GANT-61. Scale bars, 100μm. **(C)** Flow cytometry analysis of AO stained cells in panel B. **(D)** The expression of autophagic proteins in GANT-61 treated SK-N-BE(2) cells. The densitometry ratios of LC3 II/β-ACTIN**,** ATG5/β-ACTIN and BECLIN1**/**β-ACTIN were plotted as histogram (mean ± SD), *P < 0.05, **P < 0.01. (**E**) Effect of lysosomal inhibitor BafA1 on autophagic flux induced by GANT-61. SK-N-BE(2) cells were first treated with 200nM BafA1 for 30 min and then treated with 10μM GANT-61 for 4 h, 12 h, 24 h or 48 h. The LC3 II/β-ACTIN ratio at different time points was plotted as histogram (mean ± SD), *P < 0.05, **P < 0.01. **(F)** Immunofluorescence with LC3 antibody on SK-N-BE(2) cells after 48h GANT-61 treatment. Scale bars, top: 500μm, bottom: 20μm. CON, control. **(G)** Quantification of cells with a number of LC3 puncta five times higher than basal level in panel F. **P < 0.01 **(H)** SK-N-BE(2) transfected with GFP-LC3 plasmids were treated with GANT-61 for 48h. A puncta pattern of GFP-LC3 was formed after drug treatment. Scale bar,20 μm. **(I)** Quantification of cells with GFP-LC3 puncta shown in panel H, **P < 0.01. Equal loading and transfer were verified by re-probing membranes with anti-β-ACTIN antibody in Western blot analysis. (JPEG 824 KB)

Additional file 3: Figure S3: Effects of autophagic inhibition on GANT-61 treated NB cells**. (A)** The effect of 3-MA on SK-N-BE(2) cell viability. Cell viability was measured by MTT assay. **(B)** Effect of 3-MA on autophagic proteins in SK-N-BE(2) cells. Western blot analysis was performed with anti-LC3, anti-BECLIN-1 and anti-ATG5 antibodies. The densitometry ratios of LC3 II/β-ACTIN, BECLIN1**/**β-ACTIN and ATG5/β-ACTIN were plotted as histogram. **(C)** Effect of 3-MA on AKT phosphorylation was examined by Western blot in SK-N-BE(2) cells treated with GANT-61. Values of P-AKT/AKT ratio were listed under p-AKT blots. **(D)** Effect of 3-MA on cell apoptosis. SK-N-BE(2) cells were treated with GANT-61 and 3-MA at the indicated concentration for 48 h. Apoptotic cells were quantitated by flow cytometry. **(E)** The effect of 3-MA on apoptotic protein expression. Western blot analysis was performed with anti-BCL-2 and anti-cleaved CASPASE3 antibodies. The BCL2/β-ACTIN and Cleaved-CASPASE3/β-ACTIN ratios were listed under blots. **(F)** ATG5 or ATG7 shRNA specifically knocked down ATG5 or ATG7, respectively, in SK-N-BE(2) cells. Western blot analysis was performed with anti-ATG5 and anti-ATG7 antibodies. The ATG-5/β-ACTIN and ATG-7/β-ACTIN ratios were listed under blots. **(G)** Knockdown of essential autophagic components ATG5 or ATG7 completely abolished GANT-61 induced autophagic production. The LC3 II/β-ACTIN ratio was listed under blots. **(H)** GANT61 caused a higher level of cleaved CASPASE3 and a lower level of BCL2 in ATG5 or ATG7 knockdown NB cells than those in scramble shRNA knockdown controls. The BCL2/β-ACTIN and cleaved-CASPASE3/β-ACTIN ratios were plotted as histogram. **(I)** Representative flow cytometry analysis of apoptosis in GANT-61 treated cells after PE-AnnexinV and 7-AAD double staining. siCON: scramble shRNA control, siATG5: ATG5 shRNA knockdown, siATG7: ATG7 shRNA knockdown. CON, control. Data are expressed as the mean ± SD. *P < 0.05,**P < 0.01, n.s., no statistical significance. (JPEG 4 MB)

Additional file 4: Figure S4: Effects of an apoptotic inhibitor on GANT-61 treated NB cells**. (A)** The effect of Z-VAD-FMK on SK-N-BE(2) cell viability. Cell viability was measured by MTT assay. Data are expressed as the mean ± SD of three independent experiments. *P < 0.05. **(B)** Western blot analysis was performed with anti-BCL-2 and anti-cleaved CASPASE3 antibodies. The BCL2/β-ACTIN and cleaved-CASPASE3/β-ACTIN ratios were listed under blots (mean ± SD), *P < 0.05. **(C)** Western blot analysis was performed with anti-LC3, anti-BECLIN-1 antibodies. The densitometry ratios of LC3 II/β-ACTIN and BECLIN1**/**β-ACTIN were listed under blots, (mean ± SD), *P < 0.05, n.s., no statistical significance. **(D)** Flow cytometry histogram of AO stained SK-N-BE(2) cells treated with the indicated drug. Con, control. Equal loading and transfer were verified by re-probing membranes with anti-β-ACTIN antibody in Western blot analysis. CON, control. (JPEG 360 KB)

## Abbreviations

3-MA: 3-methyladenine
AO: Acridine orange
Atg5: Autophagy related gene5
AVOs: Acid vesicular organelles
BafA1: Bafilomycin A1
Bcl-2: B cell lymphoma leukemia-2
CON: Control
DMEM: Dulbecco’s modified eagle medium
DMSO: Dimethylsulfoxide
Dhh: Desert hedgehog
FBS: Fetal bovine serum
FITC: Fluorescein isothiocyanate
Hh: Hedgehog
Ihh: Indian hedgehog
LC3: Microtubule-associated proteins light chain 3B
MDC: Monodansylcadaverine
MTT: 3-(4,5-Dimethylthiazol -2-yl)-2,5-diphenyltetrazolium bromide
NB: Neuroblastoma
NC: Nitrocellulose
PI: Propidium iodide
PVDF: Polyvinylidene fluoride
PBS: Phosphate buffer saline
Ptch: Patched
Smo: Smoothened
SDS: Sodium dodecyl sulfate
Shh: Sonic hedgehog.
