# Supplementary figures and images for: Inhibition of autophagy potentiates the efficacy of Gli inhibitor GANT-61 in MYCN-amplified neuroblastoma cells
Source: BMC Cancer. 2014 Oct 17;14:768. doi: 10.1186/1471-2407-14-768 (PMC4210511; doi:10.1186/1471-2407-14-768)

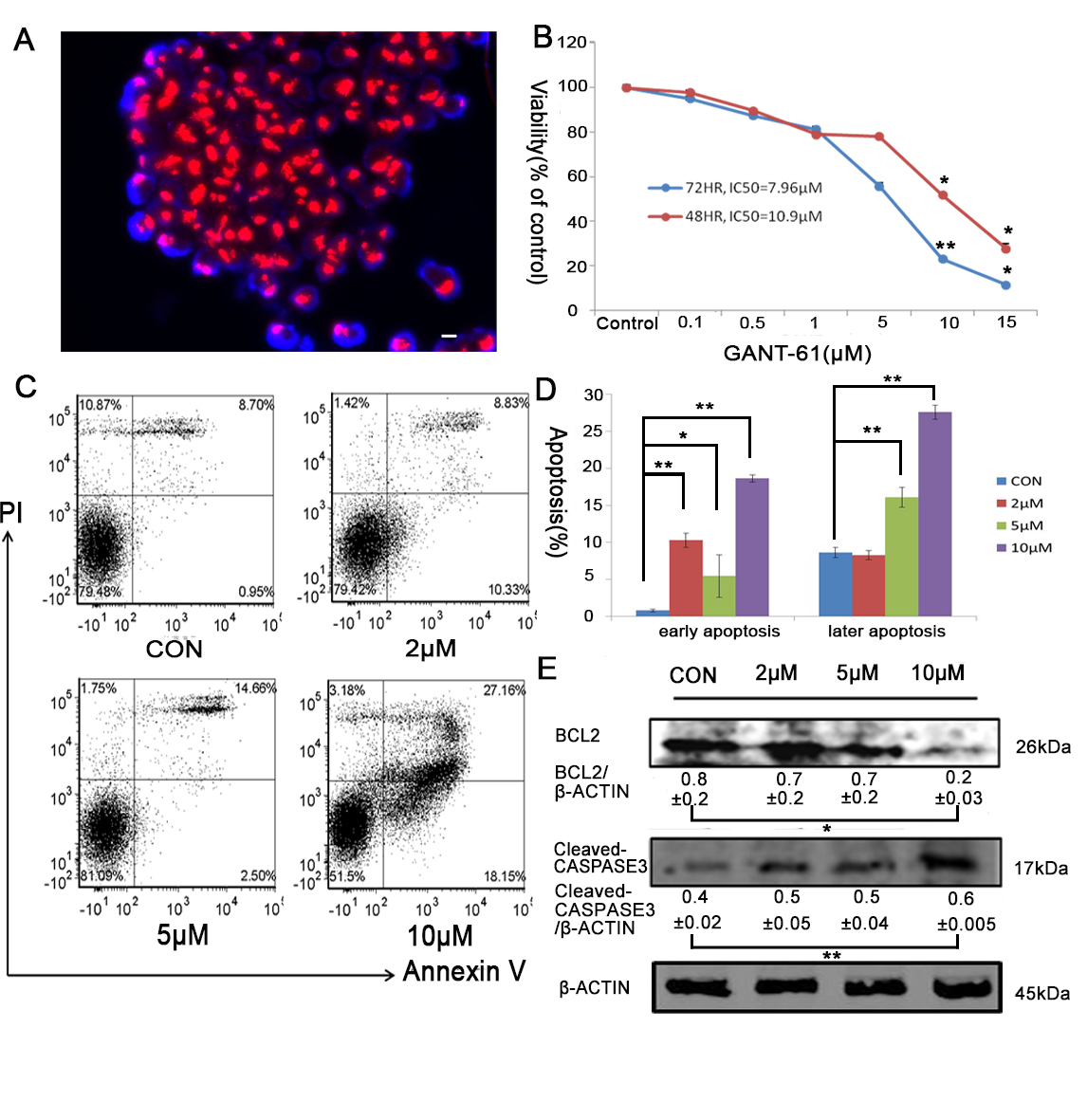

Supplement: Supplementary file 1 — Additional file 1: Figure S1: GANT-61 induces cell cytotoxicity and apoptosis in NB cells. (A) MYCN amplification was evaluated by FISH analysis in SK-N-BE(2) cells. Scale bars, 10μm. (B) GANT-61 dosage response curves of SK-N-BE(2) cells were determined using a MTT assay. The percentage of viable cells was calculated as a ratio of treated to control cells. Data is expressed as the mean ± SD of three independent experiments. *P < 0.05, **P < 0.01. (C) Flow cytometry analysis of apoptosis after AnnexinV and PI-double staining SK-N-BE(2) cells were treated with indicated concentration of GANT-61 for 48h. (D) Histogram of flow cytometry analyses from 3 independent experiments. *P < 0.05, **P < 0.01, CON, control. (E) Western blot analysis was performed to detect the expression of apoptosis-related proteins. SK-N-BE(2) cells were treated with indicated concentration of GANT-61 for 48h. The BCL2/β-ACTIN and Cleaved-CASPASE3/β-ACTIN ratios were determined by densitometry (mean ± SD), *P < 0.05, **P < 0.01. Equal loading and transfer were verified by re-probing membranes with anti-β-ACTIN antibody. CON, control. (JPEG 520 KB) [file 12885_2014_4946_MOESM1_ESM.jpeg]

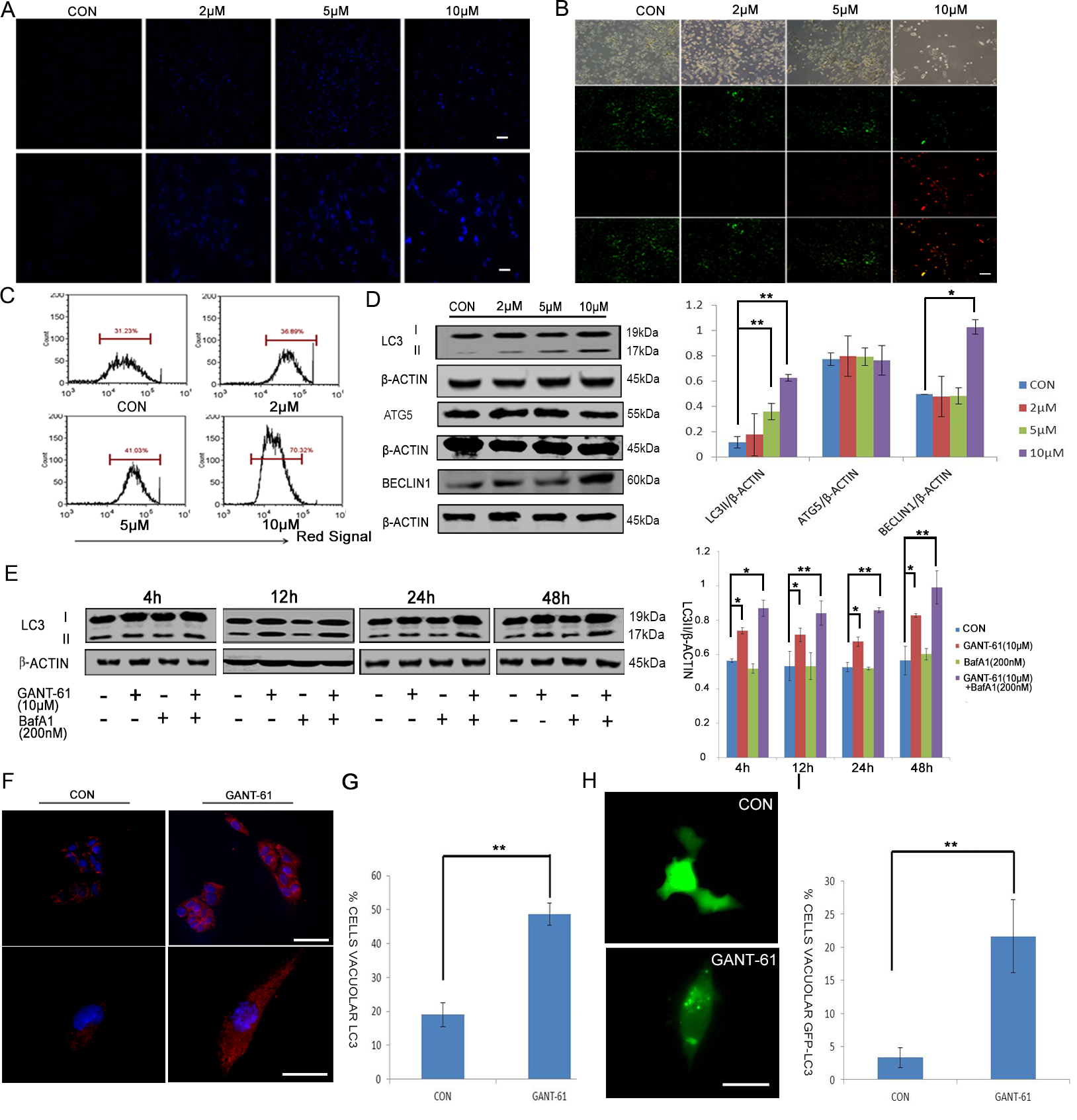

Supplement: Supplementary file 2 — Additional file 2: Figure S2: GANT-61 induces autophagy in NB cells. (A) MDC staining showed that the autophagy was activated in SK-N-BE(2) cells after GANT-61 treatment for 48h. Scale bars, top: 100μm, bottom: 50 μm. (B) Fluorescence microscopy of AO stained SK-N-BE(2) cells treated with the indicated concentration of GANT-61. Scale bars, 100μm. (C) Flow cytometry analysis of AO stained cells in panel B. (D) The expression of autophagic proteins in GANT-61 treated SK-N-BE(2) cells. The densitometry ratios of LC3 II/β-ACTIN, ATG5/β-ACTIN and BECLIN1/β-ACTIN were plotted as histogram (mean ± SD), *P < 0.05, **P < 0.01. (E) Effect of lysosomal inhibitor BafA1 on autophagic flux induced by GANT-61. SK-N-BE(2) cells were first treated with 200nM BafA1 for 30 min and then treated with 10μM GANT-61 for 4 h, 12 h, 24 h or 48 h. The LC3 II/β-ACTIN ratio at different time points was plotted as histogram (mean ± SD), *P < 0.05, **P < 0.01. (F) Immunofluorescence with LC3 antibody on SK-N-BE(2) cells after 48h GANT-61 treatment. Scale bars, top: 500μm, bottom: 20μm. CON, control. (G) Quantification of cells with a number of LC3 puncta five times higher than basal level in panel F. **P < 0.01 (H) SK-N-BE(2) transfected with GFP-LC3 plasmids were treated with GANT-61 for 48h. A puncta pattern of GFP-LC3 was formed after drug treatment. Scale bar,20 μm. (I) Quantification of cells with GFP-LC3 puncta shown in panel H, **P < 0.01. Equal loading and transfer were verified by re-probing membranes with anti-β-ACTIN antibody in Western blot analysis. (JPEG 824 KB) [file 12885_2014_4946_MOESM2_ESM.jpeg]

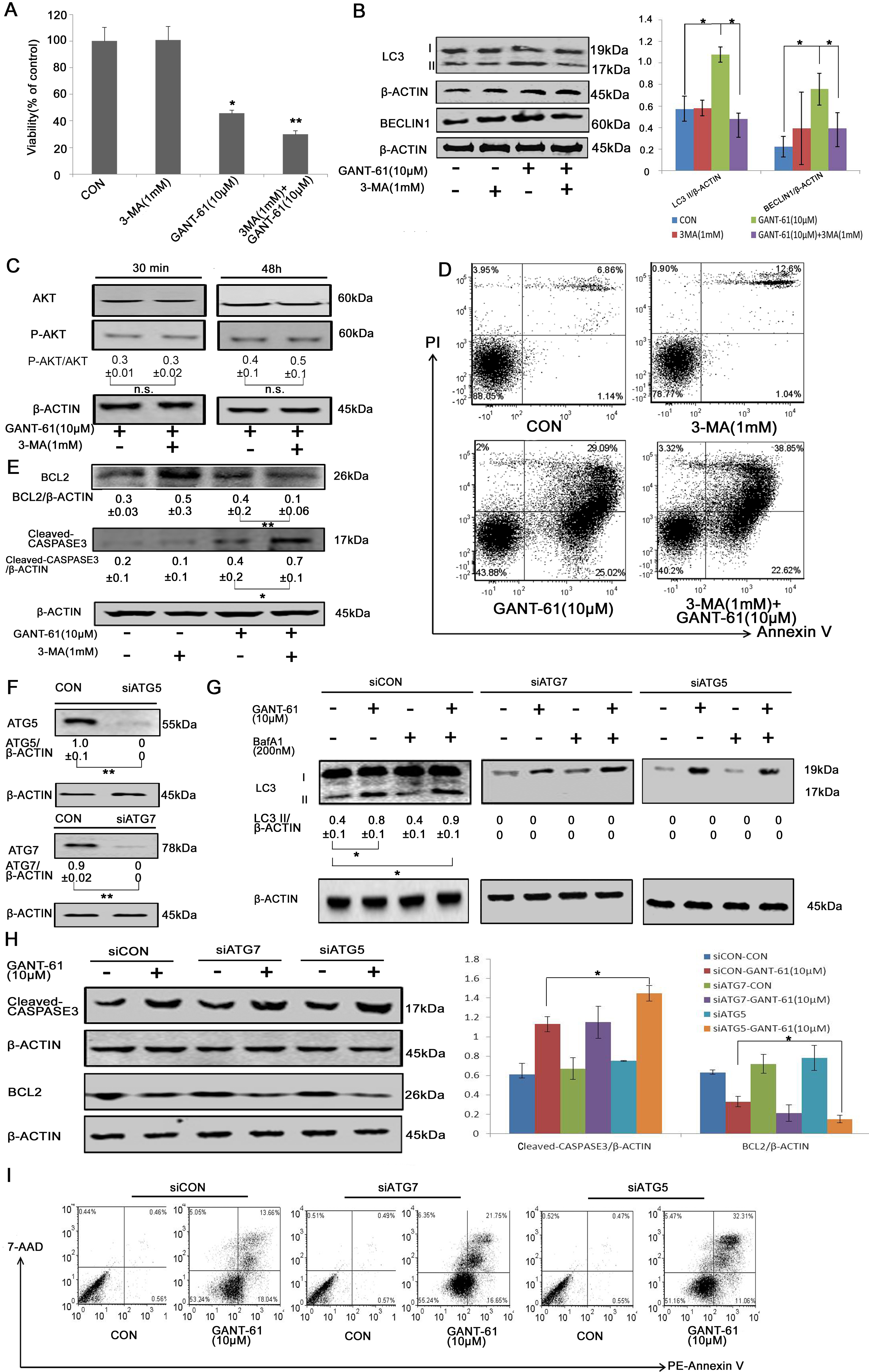

Supplement: Supplementary file 3 — Additional file 3: Figure S3: Effects of autophagic inhibition on GANT-61 treated NB cells. (A) The effect of 3-MA on SK-N-BE(2) cell viability. Cell viability was measured by MTT assay. (B) Effect of 3-MA on autophagic proteins in SK-N-BE(2) cells. Western blot analysis was performed with anti-LC3, anti-BECLIN-1 and anti-ATG5 antibodies. The densitometry ratios of LC3 II/β-ACTIN, BECLIN1/β-ACTIN and ATG5/β-ACTIN were plotted as histogram. (C) Effect of 3-MA on AKT phosphorylation was examined by Western blot in SK-N-BE(2) cells treated with GANT-61. Values of P-AKT/AKT ratio were listed under p-AKT blots. (D) Effect of 3-MA on cell apoptosis. SK-N-BE(2) cells were treated with GANT-61 and 3-MA at the indicated concentration for 48 h. Apoptotic cells were quantitated by flow cytometry. (E) The effect of 3-MA on apoptotic protein expression. Western blot analysis was performed with anti-BCL-2 and anti-cleaved CASPASE3 antibodies. The BCL2/β-ACTIN and Cleaved-CASPASE3/β-ACTIN ratios were listed under blots. (F) ATG5 or ATG7 shRNA specifically knocked down ATG5 or ATG7, respectively, in SK-N-BE(2) cells. Western blot analysis was performed with anti-ATG5 and anti-ATG7 antibodies. The ATG-5/β-ACTIN and ATG-7/β-ACTIN ratios were listed under blots. (G) Knockdown of essential autophagic components ATG5 or ATG7 completely abolished GANT-61 induced autophagic production. The LC3 II/β-ACTIN ratio was listed under blots. (H) GANT61 caused a higher level of cleaved CASPASE3 and a lower level of BCL2 in ATG5 or ATG7 knockdown NB cells than those in scramble shRNA knockdown controls. The BCL2/β-ACTIN and cleaved-CASPASE3/β-ACTIN ratios were plotted as histogram. (I) Representative flow cytometry analysis of apoptosis in GANT-61 treated cells after PE-AnnexinV and 7-AAD double staining. siCON: scramble shRNA control, siATG5: ATG5 shRNA knockdown, siATG7: ATG7 shRNA knockdown. CON, control. Data are expressed as the mean ± SD. *P < 0.05,**P < 0.01, n.s., no statistical significanc [file 12885_2014_4946_MOESM3_ESM.jpeg]

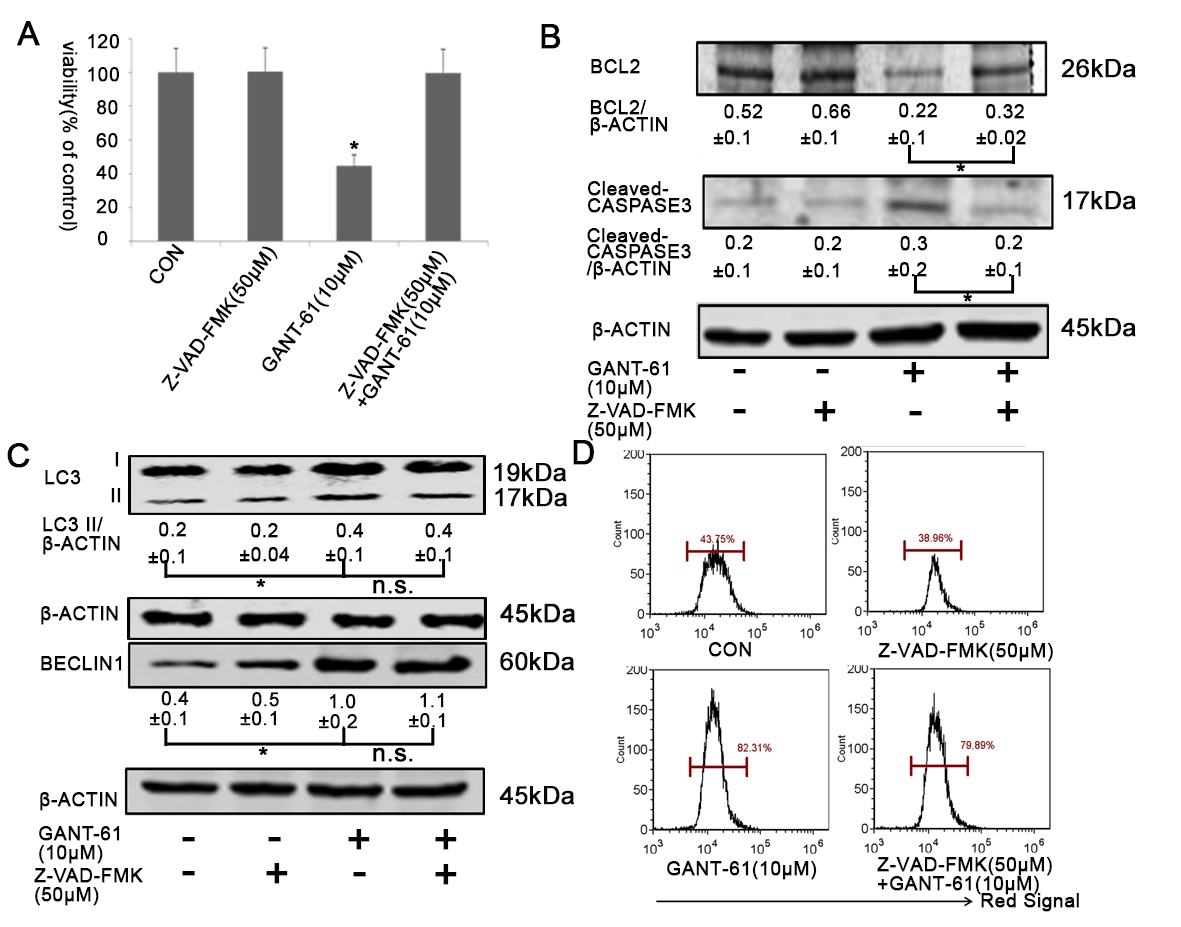

Supplement: Supplementary file 4 — Additional file 4: Figure S4: Effects of an apoptotic inhibitor on GANT-61 treated NB cells. (A) The effect of Z-VAD-FMK on SK-N-BE(2) cell viability. Cell viability was measured by MTT assay. Data are expressed as the mean ± SD of three independent experiments. *P < 0.05. (B) Western blot analysis was performed with anti-BCL-2 and anti-cleaved CASPASE3 antibodies. The BCL2/β-ACTIN and cleaved-CASPASE3/β-ACTIN ratios were listed under blots (mean ± SD), *P < 0.05. (C) Western blot analysis was performed with anti-LC3, anti-BECLIN-1 antibodies. The densitometry ratios of LC3 II/β-ACTIN and BECLIN1/β-ACTIN were listed under blots, (mean ± SD), *P < 0.05, n.s., no statistical significance. (D) Flow cytometry histogram of AO stained SK-N-BE(2) cells treated with the indicated drug. Con, control. Equal loading and transfer were verified by re-probing membranes with anti-β-ACTIN antibody in Western blot analysis. CON, control. (JPEG 360 KB) [file 12885_2014_4946_MOESM4_ESM.jpeg]
